# Our Mecca in 1900

**Published:** 1899-10

**Authors:** 


OF the three cities named, where will be our Mecca in 1900—Minneapolis, Milwaukee, or Detroit? All are good cities; central Western points in the Association's membership. Minnesota's strong membership in our organization, her large percentage in attendance the last five years, her splendidly directed veterinary sanitary control work, are weighty claims on the Association for the place of meeting in 1900.

